# Self-Organizing Traffic Flow Prediction with an Optimized Deep Belief Network for Internet of Vehicles

**DOI:** 10.3390/s18103459

**Published:** 2018-10-15

**Authors:** Shidrokh Goudarzi, Mohd Nazri Kama, Mohammad Hossein Anisi, Seyed Ahmad Soleymani, Faiyaz Doctor

**Affiliations:** 1 Advanced Informatics School, Universiti Teknologi Malaysia Kuala Lumpur (UTM), Jalan Semarak, Kuala Lumpur 54100, Malaysia; gshidrokh2@live.utm.my (S.G.); mdnazri@utm.my (M.N.K.); 2 School of Computer Science and Electronic Engineering, University of Essex, Colchester CO4 3SQ, UK; fdocto@essex.ac.uk; 3 Faculty of Computing, Universiti Teknologi Malaysia Kuala Lumpur (UTM), Skudai, Johor 81310, Malaysia; asseyed4@live.utm.my

**Keywords:** deep belief network, historical time traffic flows, restricted Boltzmann machine, optimization, traffic flow prediction

## Abstract

To assist in the broadcasting of time-critical traffic information in an Internet of Vehicles (IoV) and vehicular sensor networks (VSN), fast network connectivity is needed. Accurate traffic information prediction can improve traffic congestion and operation efficiency, which helps to reduce commute times, noise and carbon emissions. In this study, we present a novel approach for predicting the traffic flow volume by using traffic data in self-organizing vehicular networks. The proposed method is based on using a probabilistic generative neural network techniques called deep belief network (DBN) that includes multiple layers of restricted Boltzmann machine (RBM) auto-encoders. Time series data generated from the roadside units (RSUs) for five highway links are used by a three layer DBN to extract and learn key input features for constructing a model to predict traffic flow. Back-propagation is utilized as a general learning algorithm for fine-tuning the weight parameters among the visible and hidden layers of RBMs. During the training process the firefly algorithm (FFA) is applied for optimizing the DBN topology and learning rate parameter. Monte Carlo simulations are used to assess the accuracy of the prediction model. The results show that the proposed model achieves superior performance accuracy for predicting traffic flow in comparison with other approaches applied in the literature. The proposed approach can help to solve the problem of traffic congestion, and provide guidance and advice for road users and traffic regulators.

## 1. Introduction

**Figure 1:**
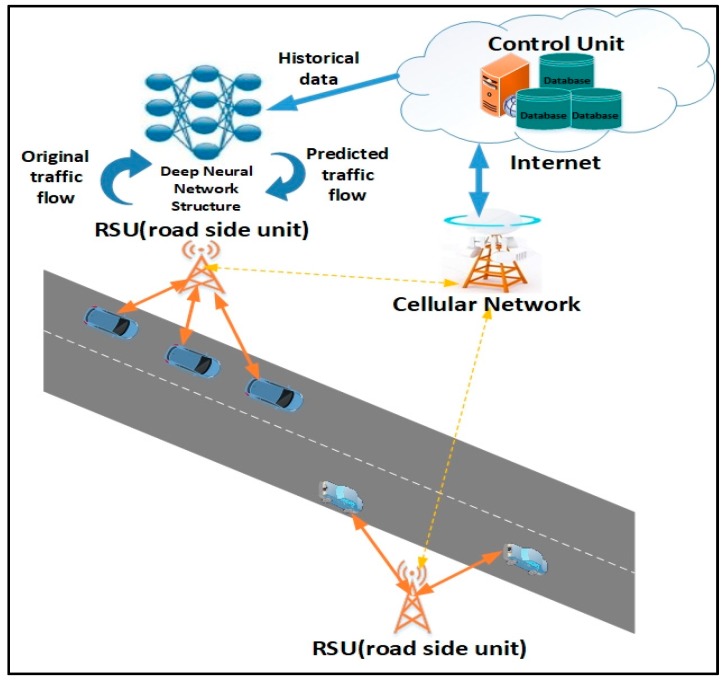
Delivery of content to vehicles via vehicle-to-roadside (v2r) links.

In an IoV and VSN, vehicles act as senders, receivers and routers to broadcast data to a network or transportation agency as part of an integrated Intelligent Transportation System (ITS) [1]. The collected data can be used for traffic flow prediction to ensure safe, free-flow of traffic in metropolitan areas. The application of sensor networks as a roadside communication infrastructure is regularly used in various current intelligent transportation and smart highway systems. The roadside units (RSUs) offer a secure infrastructure along the road which are responsible for broadcasting periodic safety messages to road users. Typically, RSUs are located every 300 m to 1 km and transmit data at the interval of every 300 ms. Therefore, placing RSUs along a long stretch of highway to offer ubiquitous connectivity is not economically viable. Hence, vehicles should be able to use other vehicles to transmit and receive driver critical data feeds with limited support from fixed road side infrastructures [1,2]. In this paper, we developed smart prediction scheme for vehicle-to-vehicle (V2V) communication [3,4], where the vehicles can obtain predicted information using their on-board units (OBUs) which is computed by RSUs. A basic scenario of content delivery to vehicles at various ranges through vehicle-to-roadside (V2R) links is presented in Figure 1. The main operational functions of the real time prediction system depicted includes: traffic data archiving, traffic pattern processing and traffic flow forecasting. Traffic data would be collected by RSUs for purposes of data analysis. Traffic pattern processing would create a dynamic traffic pattern (TP) matrix using the collected data to assess traffic volume. This paper focuses on developing the traffic flow forecasting unit which uses the TP matrix for constructing a traffic flow prediction model.

The main challenge is that short-term traffic estimations may be inaccurate because of unpredictable disruptions such as accidents on the road. Historical traffic flow data should be used for traffic time estimation in a network. Nevertheless, activity time forecast cannot exclusively depend on past movement information because of the following reasons: (1) On-street disruptions and accidents which would affect traffic flows in the network, the impact of which cannot be anticipated; (2) off-road events can affect traffic flows and they cannot be incorporated into the typical historical traffic time information; and finally, (3) traffic information is not accessible for all connections in a traffic network due to the fact that most connections are not equipped with traffic sensors.

Accurately anticipating traffic time is an imperative element of IoV and intelligent transportation frameworks [5,6]. There are a wide range of traffic time prediction techniques incorporating time arrangement examination [7,8], Bayesian systems [9], neural networks (NNs) [10,11,12,13], fuzzy systems [2], fuzzy NNs [14,15], nonparametric regression (NP) [16,17], and other computational intelligence approaches [18]. The availability of travel time data is increasingly being used for modelling traffic behaviour to assist road users and city authorities to make better informed decisions about travel choices, levels of pollution and congestion, the effect on public and private transportation policies, and effective repair and maintenance of the road network. However, data can often be missing for specific timeframes due to noise in the reading or corrupted data [19,20,21,22]. Various machine learning, probabilistic and statistical modelling approaches have attempted to solve the problem of missing data in traffic forecasting [23,24,25,26,27,28,29]. A study by van Lint et al. [30] showed a travel time forecasting model based on a neural system for handling missing traffic information while in Sun et al. [31] traffic streams estimation based on using a Bayesian model was presented where missing historical traffic information was estimated by utilizing a Gaussian blend display to visually verify the traffic data forecast. Various specialists have shown that hybrid methods have better results in terms of accuracy and precision compared with individual techniques [32]. Hybrid methods based on fuzzy logic can be potential alternatives to enhance precision in traffic flow prediction as described in [33] while in [34] a novel method based on neural networks is utilized in traffic time estimation.

Artificial neural networks (ANNs) have been widely used for time series prediction problems since their inception in the 1980s. In classical neural networks, training algorithms akin to back- propagation only try to model the dependence of the output from the input. Restricted Boltzmann Machines (RBMs), instead, are networks of stochastic neurons that can be trained in a greedy fashion. Deep belief networks are obtained by stacking RBMs on one another so that the input to one layer is given by the hidden units of the adjacent layer, as if they were data, and adding a last discriminative layer. The RBM, might even yield better results than traditional neural networks with higher accuracy. In a RBN, the hidden units are independent given the visible states. So, they can quickly get an unbiased sample from the posterior distribution when given a data-vector. This is a big advantage over direct belief nets. The multi-layer perceptron (MLP) and radial basis function networks (RBFN) are well-known approaches. Often gradient descent methods are used for training these approaches and back propagation (BP) is used as the learning algorithm [27].

However, there are some limitations of using conventionally shallow ANNs for real world problems such as traffic flow prediction in highways based on VANET-cellular systems. The first issue is related to the design of the ANN topology. It is found that the larger the size of the hidden layer the more prone the model is to overfitting the training data. The second problem is related to deciding the initial value of the ANN weights. BP is a supervised learning method which uses samples of input and output data to modify weights of connections between units (neurons) across the network layers. The appropriate selection of initial weights can increase the speed with which the model is able to converge. Both these problems are amplified when the input parameter space is very large as in the case of traffic flow prediction. Hence there is a need to be able to transform the input parameters into a reduced and manageable feature space with which to construct the prediction model. Equally there is a need to determine the optimal number of hidden neurons for training the model. Finally, the third problem is determining a suitable learning rate during the models training phase. Here there is a need to incorporate an automated way of selecting the most appropriate learning rates as the model is being trained. To solve these problems, we proposed a novel traffic flow prediction model based on DBNs comprised of multiple stacked restricted Boltzmann machine (RBM) auto-encoders. RBMs are networks of stochastic units with undirected interactions between pairs of visible and hidden units which can be used to learn a probability distribution over its set of inputs. By stacking multiple RBMs onto one another DBNs are trained using greedy layer-wise learning which aims to train each layer of a DBN in a sequential and unsupervised way, feeding lower layer results to the upper layers to capture a representational hierarchy of relationships within the training data [10,11,12,13]. Each trained layer represents feature encoders which can be helpful in the discrimination of the target output space. This unsupervised training process can provide an optimal start for supervised training as well as extract and learn a reduced set of features representing the input parameters. Supervised learning is then performed using backpropagation for fine-tuning the weight parameters among the visible and hidden layers of RBMs for training the traffic flow prediction model. The firefly Algorithm (FFA) is further applied for selecting the optimal number of connected units (neurons) and learning rate during training of the proposed model which has been termed DRBM-FFA. In brief, the main contribution of this study can be listed as follows:

- We define a dynamic traffic pattern matrix to assess traffic volume data;
- We propose a 3-layer DBN composed of two RBMs to determine the salient features from time series traffic volume data for constructing a traffic flow prediction model on VANET-cellular systems.
- We utilize FFA algorithm to optimize and select the sizes of the learning rates in neural networks and;
- We perform simulations and explain how to use historical traffic data for traffic volume prediction.

The reminder of this paper is organized as follows: Section 2 shows the initial Traffic Pattern (TP) matrix to assess traffic time data at five highway links. A dynamic (TP) matrix predictor based on a DBN of RBMs is presented in Section 3. The (FFA) algorithm for selecting the best number of units and for selecting the rates of learning of deep belief nets is explained in Section 4. We demonstrate our predictions and results in Section 5 and the conclusions in Section 6. 

## 2. Assessing Traffic Pattern Matrix

This section focuses on the effective procedure to predict traffic pattern in vehicular communications for utilization in real-time applications, such as dynamic traffic management. RSUs can collect speed and flow data and the information gathered can be delivered to a control unit that automatically estimates volume of traffic [35]. 

The pattern of traffic can be characterized as a matrix on a temporal and spatial scale. The spatial scale incorporates the entire area of the street for which specific trip times can be anticipated. The temporal scale incorporates adequate time spans to characterize the impact of traffic on travel time. Traffic volume is specified as the number of vehicles that cross a section of road per unit time within a selected period. Volume of traffic can influence travel time together with speed of vehicles which is utilized as a marker for congestion. We assigned the weights at given times and locations to create the TP matrix based on congestion level to optimize travel times. The principal task here is to derive a historical days’ database by using the assumption that traffic patterns are repetitive during a tight time period, for example, traffic time for 10 a.m. traffic can be viewed from 9 a.m. to 11 a.m. This search window can locate comparative traffic patterns rapidly. There are traffic examples of different days which are recorded in the database inside a time span of ±x minutes for time estimation. Our scenario links to V2R communications and measures vehicles moving at speeds of 100 km/h (~27 m/s) crossing each of the RSU with a coverage range of 200 m (radius). This relates to the high contact duration of 200 × 2/27 ≈ 15 s. 

In our simulation, we assumed that the road section consists of *k* links and each link shows a section of road. Each section should be equipped with one RSU, the amount of days in historical database is denoted by $i=1,2,3,\;\ldots n_{i}$, $j=0,5,10,\ldots ,n_{j}$ representing information in a five minutes resolution, and t which is the prediction time on prediction day p. The start time of the traffic pattern on historical days denoted by $t_{s}.\;v\left(i,\;t-j,p\right)$ designates velocity on prediction day p at link k at time t−j. Similarity, $h=1,2,3,\ldots ,n_{h}$ shows the number of days in historical database and $t_{s}$ shows the beginning time of the traffic pattern on historical days then $v\left(i,t_{s}-j,h\right)$ shows on historical day h at link k at time $t_{s}-j$. Travel time on a road is mainly affected by the congestion present on the road. This congestion may occur due to bottlenecks. Weights are applied to account for the congestion produced due to the type of bottlenecks, whenever and wherever it occurs. We set the weights according to the rapid speed of each section. These weights have to be higher for the sections with lower rapid speeds which represented bottlenecks. The following practical formula is utilized in Equation (1):

(1) $$w\left(i,j\right)=\frac{1}{{\left[v\left(i,t-j,p\right)\right]}^{C}}$$

where, *C* is a constant. The search is executed in ±x minutes of estimation time t on historical days so $t+x\geq t_{s}\geq t-x$. The basic purpose of the pattern matching process is to find the most similar historical pattern(s). Hence, the primary task is to generate some historical days’ database. One way of searching these patterns is to discover the entire historical database for the most similar pattern, but this makes the search process computationally intensive. Hence, the sum of the squared difference between the prediction time traffic pattern and the historical traffic patterns is used as a criterion for finding similarities between the traffic patterns. The historical traffic pattern having minimum sum of squared difference, is regarded as the most similar pattern. The objective function formula for forming the traffic patterns can be determined by Equation (2):

(2) $$\Delta^{2}\;\left(p,t,h,t_{s}\right)=\overset{n_{i}}{\underset{i=0}{\sum }}\overset{n_{j}}{\underset{j=0}{\sum }}w\left(i,j\right).\frac{L\left(i\right)}{L}\;{\left[\frac{1}{v\left(i,t-j,p\right)}-\frac{1}{v\left(i,t_{s}-j,\;h\right)}\right]}^{2}$$

where the traffic weight in cell (*i, j*) is shown by w(i,j), length of section *i* is presented by L(i) and the stretch length of the road is shown by *L* and $\Delta^{2}\;\left(p,t,h,t_{s}\right)$ denotes the squared difference among the current and historical pattern. After assigning the TP matrix, standard deviation, the coefficient of determination $R^{2}$, the mean square error and linear regression line parameters should be determined. The TP matrix fixes the trip’s numbers with zones in each short period of time. Each TP matrix is allocated to each transportation option. Each link shows streets and highways and nodes which can be connected by links. The Table 1 shows values for the highway links.

**Figure 2:**
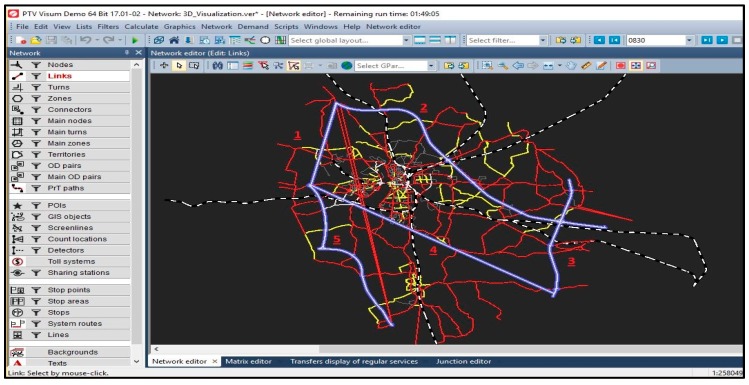
Case study traffic network with five highways links. The numbers 1 to 5 illustrate 5 highways links.

A commercial software called PTV Visum [36] is used to simulate a traffic road network. The software is used for multimodal transportation planning with an integrated network model for private and public transport. The TP matrix is used as inputs to the PTV Visum simulation, and the outputs are the predicted traffic volume. The TP matrix is assigned according to the available traffic volumes. The input information from the PTV Visum [37] offers a guideline for the traffic flow completion model. Traffic information are collected each minute for five of the links. Figure 2 depicts a screenshot of the simulation showing connections 1–5 that are the highway links.

## 3. DBN for Time Series Forecasting

Machine learning modelling based on DBN has emerged as a technique to improve measurement data. DBNs are deep neural network models comprising of multiple layers of hidden nodes representing latent variables for detecting features extracted from the original multi-dimensional input data [38]. These models can be trained in a generative unsupervised manner where the model learns to probabilistically reconstruct the inputs from abstracted features extracted at each layer [38]. Following this learning step the DBN can be further trained as a discriminative supervised learning model to perform classification or time series prediction. There are three main reasons for using DBN as follows: They take numerous non-linear hidden layers, have the ability to be pre-trained in an unsupervised manner and allow the hidden state to be factored in an arbitrary way.

The traffic prediction algorithm is designed based on current and historical traffic flows data derived from a database of RSUs. We propose a strategy that predicts the activity time for every one of the 5 highways links over a brief time horizon in a transportation network which comprises of two stages: (1) traffic time information fulfilment and (2) Short-term traffic flow forecasting. In stage 1, trip distribution estimation is derived from the TP matrix to create traffic time information at each link based on demand and recorded information in the initial TP matrix. In stage 2, we utilize the traffic volume data at each link produced from stage 1 to anticipate traffic flow recursively by a network using two RBMs by adjusting in historical information to account for unpredictable changes. In this study, we designed a three-layer DBN constructed by using two stacked RBMs [25,26] to propose the traffic flow time series prediction model.

When high dimension data are input to the units of visible layer of an RBM, the units in the RBM’s hidden layer detects the feature of data among different classes according to the connection weights. The connection of units of RBM is restricted to different layers, which means that no connections exits between the units of same layer, so the paired layers are termed as a restricted Boltzman machine. When the hidden layer of one RBM is used as a visible input layer for a second RBM, the second RBM’s hidden layer determines “the feature of features” of the original input data. Therefore, the two stacked RBMs are able to determine a restricted set of features derived from the original higher dimensional input parameters. 

**Figure 3:**
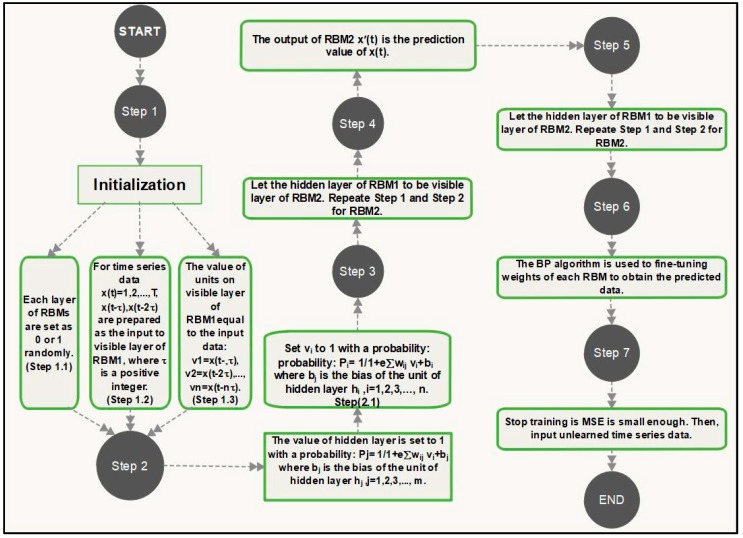
Steps of (DBN) with two (RBMs).

In the initial step of the training process, the data units for each layer are set randomly to values of 0 or 1. For training the algorithm we randomly selected 30 training instances and evaluated the model on 30 test instances respectively. The weights $w_{ij}$ among data units for each layer are set to values between 0 and 1. There is no connections between units of each layer of RBMs. The input units $v_{i}$ of visible layer of RBMs are shown as x(t−α), x(t−2α),…, x(t−nα) for input data x(t), t=1,2,3,…,T. We calculate the expectation of data by $p_{ij}^{data}=\langle x\left(t-i\alpha \right)h_{j}\rangle$. Then, we calculate the expectation for reconstruction by $p_{ij}=\langle v_{i}h_{j}\rangle$, where $h_{j}$ refers to the values of unites in the hidden layer of the RBM, α is a positive integer and $v_{i}$ is the binary state of input x(t), i=1,2,3,…, n where n is defined as the dimension of the input data which shows the number of units on visible layer of RBMs. After this initial step, the weights $w_{ij}$ should be updated by $\Delta w_{ij}=\beta \left(p_{ij}^{data}-p_{ij}\right)$, where β is a rate of learning (0 < β<1). The hidden layer of first RBM then feeds in as the visible layer of second RBM. When the visible layer of each RBM receives the higher dimension information as inputs, the respective hidden layers classify the components of information among various classes using association weights. There is a limitation of association between units of each layer, so the matched layers are considered as RBM [27,39]. The hidden layer of the second RBM further evaluates the classified information to extract a reduced set of features. This initial training step ensures that RBM weights are approximated close to the ideal solution. The back-propagation (BP) algorithm [40] is then used for fine-tuning the weights of each RBM to get a refined prediction. Here the loss function used to evaluate the model’s performance is based on the mean squared error (MSE) among x(t) and x(tn−α). The MSE is considered as a stopping criteria based on whether the MSE is small enough MSE < ε, where ε is a small and positive parameter. The stages of the model training process are shown in Figure 3.

**Figure 4:**
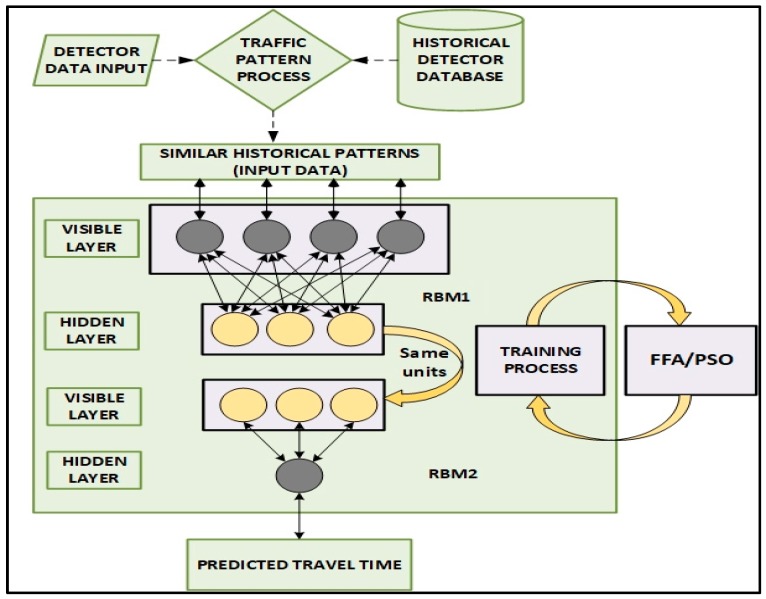
Proposed DRBM-FFA prediction model.

In order to optimize DBN model further the number of units in the RBMs visible and hidden layers together with the learning rate are optimized by FFA according to the characteristic for neural network prediction models [41,42]. Figure 4 displays the diagram of the proposed DRBM-FFA modelling framework. Here the traffic detector data is utilized to describe present traffic patterns that is found in the historical database, whereby *n* best comparable patterns are chosen from historical data to derive the current traffic state and anticipate travel time using the proposed model.

## 4. Optimization of the DBN Prediction Model

Generally, the number of layers, number of units on input layer, hidden layer and learning rate should be optimized for designing an effective neural network model. Our model utilizes a three-layer DBN with the adoption of FFA [43] to find the learning rate of RBM, number of units on input layer and number of units on both hidden layers for RBM1 and RBM2 as shown in Figure 4.

### Optimization by Firefly Algorithm (FFA)

This section clarifies the structure of the DBN parameter’ optimization utilizing the FFA. The quantity of layers, the quantity of units within each layer, and the rate of learning of the DBN should be chosen to accurately model volume traffic flow prediction. The FFA is a nature-inspired optimization method based on the social mating behaviour of fireflies [43]. This algorithm belongs to the class of swarm intelligence techniques that is based on the bioluminescence flashing behaviour of fireflies, which acts as a signaling system to attract other fireflies and was developed by Yang [44]. In this algorithm each firefly can flash with some degree of brightness. This light can be attractive for other neighboring fireflies and their attraction is influenced by the distance between fireflies. Two fireflies which are close together will a have higher attraction to each other. Each firefly symbolizes a point in a search space and the objective function is denoted by the attractiveness degree of each firefly. The fireflies should move towards their neighbours with the highest attraction. There are two essential parts to FFA: the difference of light intensity, and the definition of their engaging quality. It is considered that the attraction of each firefly is measured by its light intensity. The measurement is related to the encoded objective function. Note that the light intensity *L*(*d*) differs with the distance *d*, based on Equation (3):

(3) $$L=L_{0}e^{-\gamma^{d^{2}}}$$

where intensity of light and value of the absorption coefficient are displayed by $L_{0}$ and γ separately. As a firefly’s attractive quality corresponds to the light intensity realized through neighboring fireflies. Then, we would be able to characterize the attraction *β* of a firefly as being:

(4) $$\beta =\beta_{0}e^{-\gamma^{d^{2}}}$$

where $\beta_{0}$ is the attraction at d=0. The distance among any two fireflies i and j at $X_{i}$ and $X_{j}$ can be the Cartesian distance $d_{ij}$ = ${\left\|X_{i}-X_{j}\right\|}^{2}$ or the 2-norm. 

If a firefly *i* is attracted to another brighter firefly *j* and firefly i is moving towards j, then the movement of a firefly *i* can be measured by following equation:

(5) $$X_{i}=X_{i}+\beta_{0}e^{-\gamma^{d^{2}}}\left(X_{j}-X_{i}\right)+\alpha \varepsilon_{i}$$

where the second term will also contribute to the intensity of attraction and the third term comes from a Gaussian distribution which is presented by the vector of random variables $\varepsilon_{i}$. 

We utilized the FFA to choose the best number of units per layer of RBM, assuming the prediction model is using two stacked RBMs as shown in Figure 4. The visible and hidden layers of the first RBM have *n* and *m* units respectively. The visible layer of the first RBM is similar to the visible layer of the second RBM. The hidden layer of the second RBM has 1 unit that is the output of the prediction model. Given the rate of learning of RBM is denoted ε, a firefly is intended to be represented as a vector $X_{i}$ denoted as a three-dimension vector X(n;m;ε), where *n* = 1,2, …, *n*; and *m* = 1,2, …, *m*; and *ε* ∈ (0,1). Using a population of fireflies with size *P*, the FFA algorithm can be used to improved prediction performance of DBN [13]. The FFA adopted to choose the ideal number of input and hidden units of DBN. The FFA is outlined as follows in Algorithm 1.

**Algorithm 1.** Firefly AlgorithmObjective function f(x), $X_{i}={\left(x_{1},x_{1},\ldots ,x_{1}\right)}^{T}$ Decide the population size of fireflies P and set the iteration number of I. Initialize a population $X_{i}\left(i=1,2,3,\ldots ,n\right)$ of fireflies Outline γ as light absorption coefficient While (t<Maximum Generation)   For i=1:n all n fireflies   For j=1:i all n firefiles   Light intensity $I_{i}$ at $X_{i}$ is determined by $f\left(X_{i}\right)$   Evaluate per firefly via the (MSE) among the predicted value $\hat{x}$(t) and original data x(t).   If ($I_{j}>I_{i})$   Move firefly i towards j in all d dimensions   End if   Attraction differs with distance r via $exp\left[-\gamma^{r}\right]$   Assess new solutions and update light intensity   End for j   End for i   Rank the fireflies and discover the current best   Find the best firefly with best attraction from its history End *while*


## 5. Forecasting Results

**Figure 5:**
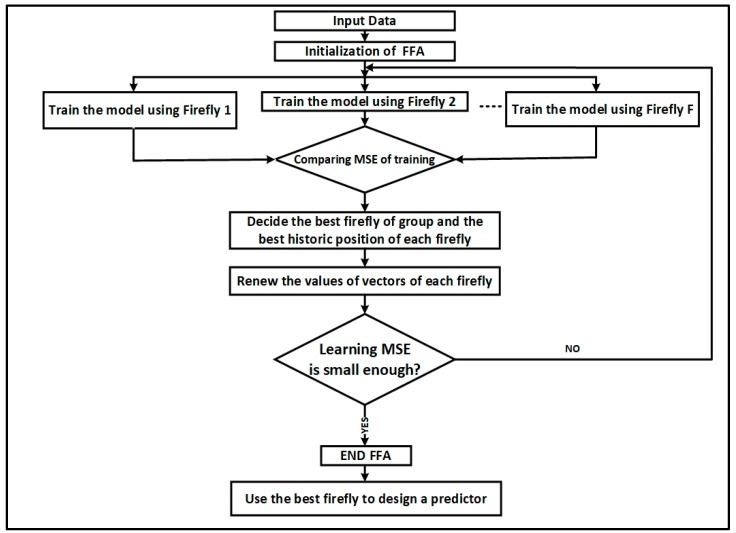
Designing an optimized predictor of DBN via the best firefly.

The time series data was used in short term prediction simulation to evaluate the performance of the proposed DRBM-FFA approach. To objectively benchmark the performance accuracy of the proposed approach it was compared with two other well know predictive modelling approaches namely a conventional Multi-Layer Perceptron (MLP) neural network and a linear ARIMA model [45]. To also have a fair evaluation of the hybrid FFA optimization strategy used, hybrid variants of MLP optimized using FFA (MLP-FFA) and ARIMA optimized using particle swarm optimization (ARIMA-PSO) were also compared with the proposed DRBM-FFA method. Figure 5 shows the designing an optimized predictor of DBN via the best firefly.

Table 2 lists the parameters and values for each of the algorithms evaluated in our prediction experiments.

**Figure 6:**
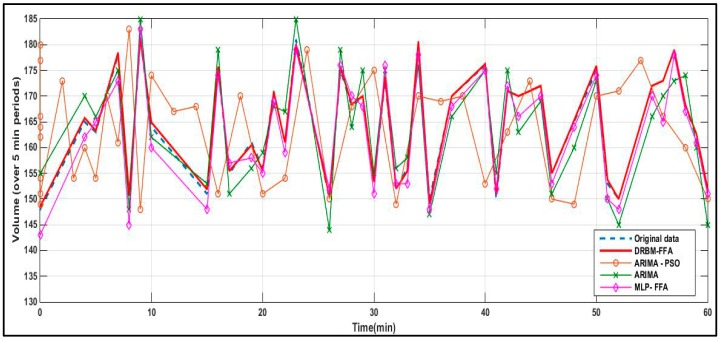
Prediction results by DRBM-FFA, ARIMA-PSO, ARIMA and MLP-FFA methods.

**Figure 7:**
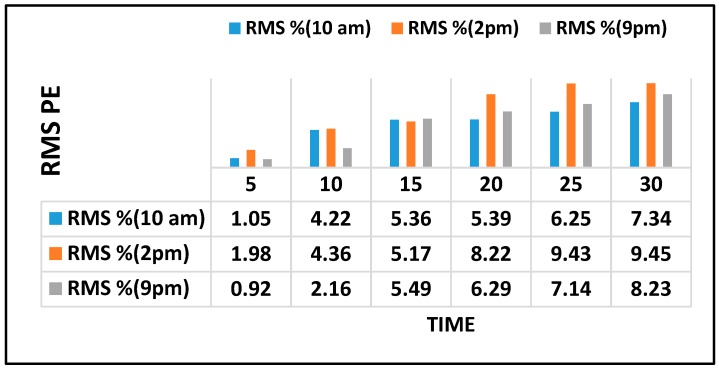
RMSPE for the targeted links.

**Figure 8:**
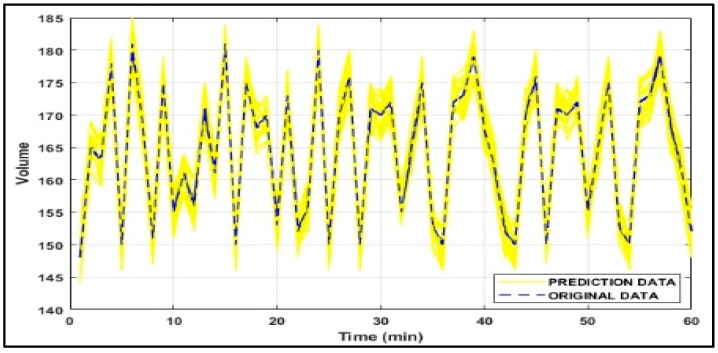
Monte Carlo experiment results over a 60-min period.

**Figure 9:**
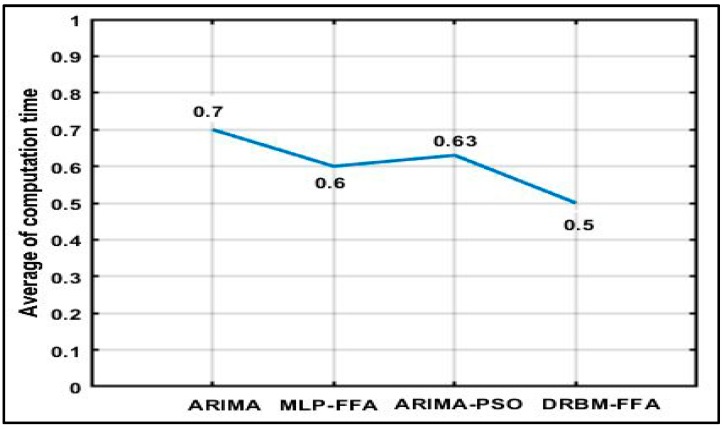
Average of computation time.

**Figure 10:**
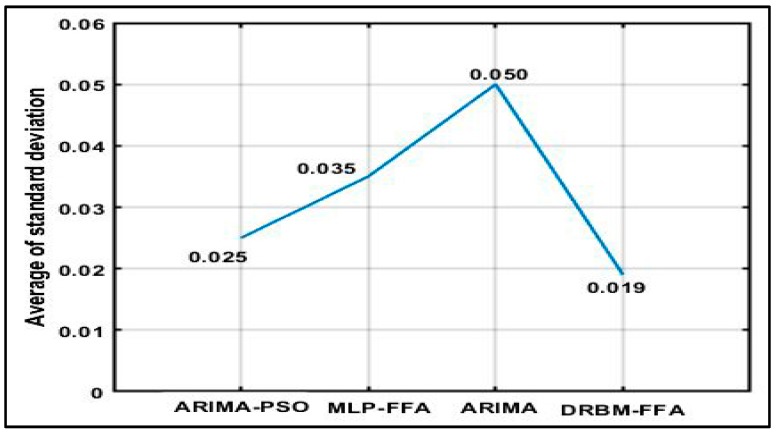
Average of standard deviation.

Short-term prediction accuracy of the DRBM-FFA model compared against the ARIMA, MLP-FFA and ARIMA-PSO are shown in Figure 6. Each algorithm is used to predict traffic flows in all five links in the traffic network where traffic data is utilized to predict traffic flow for the whole transportation network. The short-term prediction precision of the DRBM-FFA is compared against each of the other models and the results are shown in Figure 7, Figure 8, Figure 9 and Figure 10.

The flowchart of the processing steps is shown in Figure 5.

Different statistical estimators are applied to assess the performance of the proposed DRBM-FFA method. These estimators are as follows: the (MSE) shown in Equation (6), the coefficient of determination ($R^{2}$) presented in Equation (7), the root mean square (RMSE) presented in Equation (8), correlation coefficient (*r*) presented in Equation (9), mean absolute percentage error (MAPE) shown in Equation (10), root-mean square percentage error (RMSPE) displayed in Equation (11).

(6) $$\mathrm{MSE}=\frac{1}{r}\overset{r}{\underset{i=1}{\sum }}{\left(D_{pi}-D_{ai}\right)}^{2}$$

(7) $$R^{2}=1-\frac{\sum_{i=1}^{r}{\left(D_{pi}-D_{ai}\right)}^{2}}{\sum_{i=1}^{r}{\left(D_{pi}-D_{av}\right)}^{2}}$$

(8) $$\mathrm{RMSE}=\sqrt{\frac{1}{r}}\overset{n}{\underset{i=1}{\sum }}{\left(D_{pi}-D_{ai}\right)}^{2}$$

(9) $$r=\frac{\sum_{i=1}^{n}\left(D_{pi}-\bar{D_{pi}}\right).\left(D_{ai}-\bar{D_{ai}}\right)}{\sqrt{\sum_{i=1}^{n}\left(D_{pi}-\bar{D_{pi}}\right).\;\sum_{i=1}^{n}\left(D_{pi}-\bar{D_{pi}}\right)}}$$

(10) $$\mathrm{MAPE}=\frac{1}{r}\overset{n}{\underset{i=1}{\sum }}\left|\frac{D_{pi}-D_{ai}}{D_{ai}}\right|\times 100$$

(11) $$\mathrm{RMSPE}=\sqrt{\frac{1}{n}\overset{n}{\underset{l=1}{\sum }}{\left[\frac{{\bar{D}}_{pl}-D_{l}}{D_{l}}\right]}^{2}}$$

where *n* is the quantity of data, $D_{pi}$ is the predicted value; $D_{av}$ is the average of the actual values; $D_{ai}$ is the actual value; ${\bar{D}}_{pl}$ is the predicted traffic flow; $D_{l}$ shows the measured traffic flow for link l; $\bar{D_{pi}}$ and $\bar{D_{ai}}$ are the mean value of $D_{pi}$ and $D_{ai}$, respectively. The coefficient of determination, $R^{2}$ represents the linear regression line among the predicted values of the neural network model. The essential output, is applied as a measure of performance. Expressed differently, $R^{2}$ is the square of the correlation between the response values and the predicted response values. The closer $R^{2}$ is to 1, the better the model can fit the actual data [46]. This measurement controls the degree of success the fit has in stating the change of the data. It can be indicated as the square of the multiple correlation coefficients, and the coefficient of multiple determinations. The smaller amount of MAPE has a superior performance model, and conversely, in the case of r. The detail prediction errors (MSEs) for the original data are shown in Table 3.

Table 3 shows that the DRBM was able to outperform in comparison to the other approaches based on obtaining the lowest learning MSE and short-term prediction MSE based on the time series results shown in Figure 7, Figure 8, Figure 9 and Figure 10. The MLP with FFA obtained the next lowest learning MSE and short-term prediction MSE followed by the ARIMA. The Monte Carlo method was used to acquire a more objective evaluation of the performance of each approach that is based on sampling testing data based on sub-blocks to evaluate the forecasting efficiency of the algorithm.

Experiments to determine traffic flow prediction performance over five time horizons were carried out to evaluate the performance of the MLP-FFA, ARIMA, ARIMA-PSO and DRBM-FFA methods. Let P(t+1) represent the estimated flow for the (i+1)th time interval in the future. For the first forecasting interval (i+0), the flow is represented by P(t). Table 4 shows the forecasting outcomes from the test data for 5 links. In Table 4, each “t”, “t+1”, “t+2” and “t+3” is a 15-min interval into the future. The results show that the performance of all four prediction models improves when forecasting further into the future. Values in bold style show the minimum quantities for RMSE, *r* and MAPE.

Table 4 shows that all error measurement for DRBM-FFA are less than those for the other algorithms for all 15-min prediction intervals. As shown in Table 4, DRBM-FFA outperformed MLP-FFA, ARIMA, and ARIMA-PSO forecasters for all three time intervals. As anticipated, the PSO improved prediction accuracy of the ARIMA model.

Figure 7 further illustrates the prediction results of selected links every 5 min for DRBM-FFA for the next 30 min which was determined using root-mean square percentage Error (RMSPE). Figure 7 demonstrates the RMSPE for the selected links.

In addition to the given experiments, Monte Carlo [47] method is applied to assess the sensitivity and accuracy of each predictive algorithm due to the stochastic variation of traffic data. Firstly, in each experiment, the ratio of traffic flow for links is calculated. Secondly, 50% of the ratio of traffic flow for links are designated randomly. Thirdly, selected data is increased by a Gaussian random variable r. Fourthly, the new ratio of traffic flow for each link are served to the predictive method, and the results are recorded. The final stage is where, the four previous stages should be repeated 1000 times per data sample. Hence, the standard deviation of the Monte Carlo results are calculated and the coefficient r is supposed to be a Gaussian random variable r~ N(1,0.1). Figure 8 shows the error for a particular data sample for our prediction model.

Calculating the computation time (CT) taken for algorithm completion is especially necessary in the real-time IoV applications when OBU computer systems should quickly respond to any external occurrences. In this work, to obtain a fair comparison, the same computer is used for measuring the computation time (as measured by the “tic–toc” MATLAB function) [48,49]. The MATLAB R2017b on an Intel Core i5 laptop with Windows 10 system is used to carry out this measurement. In this study, the stopwatch timer functions, tic and toc, are used to calculate the computation time. Invoking tic starts the timer, and the next toc reads the elapsed time in MATLAB. The CPU time returns the total CPU time (in seconds). The line graph compares the amount of computation time on the DRBM-FFA, MLP-FFA, ARIMA, and ARIMA-PSO in 30 runs. When comparing the data resulting from the plot, the average time needed for ARIMA-PSO and MLP-FFA calculation is approximately 0.6 (s). ARIMA has a high computation time of about 0.7 (s). In contrast, DRBM-FFA has the lowest computation time of 0.5 (s). Figure 9 shows changes in the computation time between the DRBM-FFA, MLP-FFA, ARIMA, and ARIMA-PSO methods.

The standard deviation (SD) of the function values achieved in the experimental trials were also evaluated to determine prediction stability. Here the same initial conditions are used for all algorithms. The results in Figure 10 show that the standard deviation of the function values of all the other algorithms have larger values in comparison to the proposed DRBM-FFA approach. ARIMA-PSO is the next most robust approach, followed by MLP-FFA and ARIMA. The average of standard deviation for ARIMA is clearly the highest with values at 0.050, and hence their solutions qualities can be deduced to be less stable. 

In sum, the average of standard deviation obtained by DRBM-FFA is lower than those obtained by MLP-FFA, ARIMA, and ARIMA-PSO, so the proposed hybrid is very robust. Also, the swarm method of PSO achieves better result in standard deviation by ARIMA-PSO than MLP-FFA, ARIMA. The less the operating cost can be achieved with smaller standard deviations.

To further test the effectiveness of the proposed scheme, the proposed DRBM-FFA algorithm is compared in terms of complexity with the MLP-FFA, ARIMA, ARIMA-PSO algorithms. The computational complexity of the proposed model depends on the number of training samples in datasets, the structure of the RBM, the time complexity for the initialization of the fireflies, calculating the fitness values, attraction mechanism and updating the light intensity for selecting the best firefly. So, the overall computational complexity is determined as follows:

(12) $$\begin{array}{ll} O\left(\mathrm{RBM},\mathrm{FFA}\right)= & O\left(\mathrm{RBM}\right)+O\left(\mathrm{FFA}\right)\\ & =O\left(\mathrm{RBM}\right)+O\left(\mathrm{initialization}\right)+O\left(\mathrm{cost}\;\mathrm{function}\;\mathrm{calculation}\right)\\ & +O\left(\mathrm{attraction}\right)+O\left(\mathrm{light}\;\mathrm{intensity}\right)+O\left(\mathrm{best}\;\mathrm{firefly}\;\mathrm{selection}\right) \end{array}$$

The time complexity for the initialization of the fireflies is *O*(*SN*); where the *SN* is the maximum size of the population in FFA. The time complexity for calculating the fitness values is *O*(*SN*); the time complexity for updating the light intensity extreme value is *O*(*SN*); the time complexity for selecting the best firefly extreme value is *O*(*SN*). Therefore, the worst time complexity of FFA for one iteration is: *O*(*SN*) + *O*(*SN*) + *O*(*SN*) + *O*(*SN*) + *O*(*SN*), which can be simplified as *O*(FFA) = *O*(*SN*). The fixed population size (*SN*) is only considered to have an influence on the time complexity for DRBM-FFA, MLP-FFA and ARIMA-PSO models.

The overall computational complexity of an RBM depends on number of hidden units, number of the data points and number of output nodes. Additionally the computational complexity of RBM for the sequential searching takes f(T) times to get the subset of failed patterns in the best case. Therefore, the final computational complexity of the proposed method is as follows:

(13) $$O\left(\mathrm{RBM},\mathrm{FFA}\right)=O\left(SN+\left(f\left(T\right)+f\left(T\right)\times n_{h}+o\right)\right)$$

where, *T* is the number of data points, $n_{h}$ is number of hidden nodes, *o* is the number of output nodes.

For the MLP-FFA model, the computational complexity of an MLP depends on hidden nodes, number of outputs, and the number of training samples t. Given the computational complexity of FFA is O (SN), the final computational complexity of the (MLP-FFA) is as follows:

(14) O(MLP,FFA)=O(SN+(t(h+o)))

where t is the number of training samples, *h* is number of hidden nodes, *o* is the number of output nodes.

For the ARIMA-PSO model, the measure of complexity depends on the order values for *p* and *q* in ARIMA, and the time complexity of PSO algorithm. For the PSO algorithm, the time complexity can be obtained as follows: The time complexity for the initialization of the particle swarm is *O*(*SN*); the time complexity for calculating the fitness values is *O*(*SN*); the time complexity for updating the individual extreme value is *O*(*SN*); the time complexity for selecting the best individual extreme value is *O*(*SN*); the time complexity for updating the velocities and positions is *O*(*SN*). Therefore, the worst time complexity of PSO algorithm for one iteration is: *O*(*SN*) + *O*(*SN*) + *O*(*SN*) + *O*(*SN*) + *O*(*SN*), which can be simplified as *O*(*PSO*) = *O*(*SN*). The time complexity for ARIMA (*p*, *d*, *q*) depends on both *p* and *q* values [50]. Computation of AR and MA coefficients thus takes *O*((*N* − *p*) *p*2) and *O*((*N* − *q*) *q*2) time, respectively, where *N* is the length of historical values. So, the total time of the ARIMA model is as follows:

(15) $$O\left(\mathrm{ARIMA}\right)=O\left(\left(N-p\right)\times p^{2}+\left(N-q\right)\times q^{2}\right)$$

We can easily see that the complexity of ARIMA grows significantly as we consider higher order values for *p* and *q*. Therefore, the final computational complexity of the (ARIMA-PSO) is as follows:

(16) $$O\left(\mathrm{ARIMA},\mathrm{PSO}\right)=O\left(SN+\left(N-p\right)\times p^{2}+\left(N-q\right)\times q^{2}\right)$$

It can be concluded that *O*(ARIMA) > *O*(ARIMA, PSO) > *O*(MLP, FFA) ≃ (DRBM, FFA) which suggests that the hybrid approaches give an improvement in both the training and prediction process.

The effect of scalability on the computational complexity of the DRBM-FFA algorithm was also analyzed. For this purpose, time complexity of the DRBM-FFA algorithm for solving the Rosenbrock function with different dimensions was calculated as described in [51]. The Rosenbrock function was selected because it interacts between the candidate algorithms parameters. To establish the time complexity, an after-code execution time $\left(T_{0}\right)$ and the execution time of Rosenbrock function for 200,000 evaluations $\left(T_{1}\right)$ were measured and the mean of five execution times for DRBM-FFA on the Rosenbrock function using 200,000 evaluations (${\hat{T}}_{2}$) was measured. A new population is formed by the fireflies, in search of neighborhoods of the chosen solutions based on their quality. As the number of fireflies is same as *SN*, in each cycle, the fireflies carry out *SN* searches. Thus, when the maximum cycle number (*MCN*) is achieved, a total of SN×MCN searches are performed. After that, the complexity of the algorithm is established using $\frac{\left({\hat{T}}_{2}-T_{1}\right)}{T_{0}}$ as shown in Table 5 which displays the increase of ${\hat{T}}_{2}$ by less than a factor of increment dimension. As a result, it is noted that the FFA algorithm time complexity is not excessively dependent on the problem dimension and scales at O (*n*). Specifically, the DRBM-FFA totally outperforms the other methods based on aspects of solution quality and running time. 

As a result, it is noted that the DRBM-FFA time complexity is not excessively dependent on the problem dimension. Specifically, the DRBM-FFA totally outperforms the others based on aspects of solution quality and the running time.

## 6. Conclusions

The precise prediction of traffic state can help to reduce traffic congestion in large metropolitan areas. In this study, we predicted short term traffic flows using links in a traffic network and traffic data. The aim of this study was to design an accurate method for traffic flow prediction over various time periods which takes into account the spatial features of a road network to determine not only the distance but also the average speed of traffic on the links. Therefore, we proposed a DRBM-FFA prediction model based on a DBN comprising two stacked RBMs using a pre-training and fine-tuning learning algorithm. This was combined with FFA for optimizing the model’s parameters against its performance on training and testing data. Historical data on road traffic flow was used to construct and evaluate the prediction model. Results showed that for all the three evaluation measures (*r*, RMSE and MAPE), the proposed model has better performance in comparison with other traffic volume prediction methods and is able to accurately predict the traffic flow.

## Figures and Tables

**Table 1:** Values for highway links.

Criteria	Data	Value
Highway free flow template	Raw data	Data on 5 mn-spaced intervals
speed	120 km/h	10 km/5 mn interval
Average link length	2 km	5 links traversed/5 mn interval
Highway Congested template	Raw data	Data on 5 mn-spaced intervals
Average speed	72 km/h	6 km/5 mn interval
Average link length	2 km	3 links traversed/5 mn interval

**Table 2:** The used parameters in the prediction experiments.

Description	Model Elements/Parameters	Quantity
Population of PSO	P	10
The number of RBM	RBM1,RBM2	2
The number of input layer	N (1 ≤ N ≤ 20)	Given by FFA
Absorption coefficient	γ	0.1
Velocity coefficient	$c_{1},c_{2}$	1.0
The number of hidden layer	M (1 ≤ M ≤ 20)	Given by FFA
The number of output	-	1
Interval of input data	τ	1
Learning rate of RBM	ε (Step 2)	Given by FFA
Learning rate of BP	-	Given by FFA
Population of FFA	P	10
Iteration times of BP	L	100 < L < 5000
Biases of units	$b_{i},b_{j}$	0.0
Convergence parameter of BP	α	0.05
Convergence parameter of RBM	β	0.0005
Convergence period of RBM	k	50

**Table 3:** The detail prediction errors (MSEs).

Structure and Evaluation	MLP-FFA	ARIMA	ARIMA-PSO	DRBM-FFA
Learning rates	0.85	0.64	0.73	0.98
Iterations	336	350	298	200
Learning MSE	109.21	122.4	108.9	98.70
Short-term prediction MSE	234.38	280.50	126.11	109.38

**Table 4:** Traffic flow prediction results.

Predictor	Time Interval	r	RMSE	MAPE
MLP-FFA	t	3.2	6.8	12.07%
	t + 1	3.5	7.2	13.95%
	t + 2	3.6	7.8	14.89%
	t + 3	3.9	7.9	15.32%
ARIMA	t	4.4	9.1	13.56%
	t + 1	4.6	9.7	15.37%
	t + 2	6.8	14.2	18.93%
	t + 3	8.5	15.7	23.24%
ARIMA-PSO	t	3.3	6.8	9.39%
	t + 1	3.4	6.9	9.89%
	t + 2	3.7	7.2	10.48%
	t + 3	3.9	7.8	11.57%
DRBM-FFA	t	2.9	6.1	8.75%
	t + 1	3.1	6.4	9.63%
	t + 2	3.4	6.9	10.31%
	t + 3	3.5	7.1	11.12%

**Table 5:** Time Complexity Comparison.

Model	D	$T_{0}$	$T_{1}$	${\hat{T}}_{2}$	$\frac{\left({\hat{T}}_{2}-T_{1}\right)}{T_{0}}$
MLP-FFA	10	0.490	0.465	2.809	4.780
	50	0.491	0.643	2.911	4.610
	100	0.493	0.720	3.108	4.800
ARIMA	10	0.389	0.509	3.142	6.722
	50	0.378	0.734	3.708	7.855
	100	0.398	0.821	3.698	7.212
ARIMA-PSO	10	0.470	0.489	2.809	4.852
	50	0.489	0.631	2.902	4.637
	100	0.489	0.715	3.212	5.090
DRBM-FFA	10	0.411	0.233	0.703	1.145
	50	0.412	0.474	1.336	2.046
	100	0.412	0.732	1.994	2.857
